# Lifestyle, genetic risk, plasma pTau217, and incident cognitive impairment in WRAP

**DOI:** 10.1002/alz.70573

**Published:** 2025-08-13

**Authors:** Rachel L. Studer, Karly A. Cody, Julie E. Oomens, Ramiro Eduardo Rea Reyes, Nathaniel A. Chin, Kimberly D. Mueller, Bruce P. Hermann, Rachael E. Wilson, Rebecca E. Langhough, Erin M. Jonaitis, Sterling C. Johnson

**Affiliations:** ^1^ Wisconsin Alzheimer's Institute School of Medicine and Public Health University of Wisconsin ‐ Madison Madison Wisconsin USA; ^2^ Wisconsin Alzheimer's Disease Research Center Department of Medicine School of Medicine and Public Health University of Wisconsin ‐ Madison Madison Wisconsin USA; ^3^ Department of Neurology and Neurological Sciences Stanford University Palo Alto California USA; ^4^ Department of Communication Sciences and Disorders University of Wisconsin ‐ Madison Madison Wisconsin USA; ^5^ Department of Neurology School of Medicine and Public Health University of Wisconsin ‐ Madison Madison Wisconsin USA

**Keywords:** ALZpath, apolipoprotein E ε4, biomarkers, cognitive impairment, lifestyle, plasma phosphorylated‐tau217, preclinical Alzheimer's disease, risk factors

## Abstract

**INTRODUCTION:**

Whether lifestyle‐based dementia risk in midlife impacts risk of incident cognitive impairment (ICI) in the presence of apolipoprotein E (*APOE*) ε4 allele or plasma pTau217 remains unknown.

**METHODS:**

In initially cognitively unimpaired participants (*N* = 1088; baseline age, M(SD) = 57.8(6.5) years) from the Wisconsin Registry for Alzheimer's Prevention (WRAP), we investigated the relationship between lifestyle‐based dementia risk (e.g., low, moderate, and high lifestyle‐based risk tertiles) and risk of ICI when accounting for *APOE*ε4 or plasma pTau217‐ALZpath using Cox regressions (median follow‐up years = 8.2(interquartile range [IQR]: 5.5–10.8)).

**RESULTS:**

Less healthy lifestyle tertiles were associated with higher risk of ICI compared to the healthiest tertile across *APOE*ε4 carriers and non‐carriers (moderate, hazard ratio [HR] = 1.58, 95% confidence interval; [CI] 1.17‐2.15, *p* = 0.003; high, HR = 2.08, 95% CI 1.56‐2.76, *p* < 0.001) and across all pTau217 levels (moderate, HR = 1.63, 95% CI 1.20‐2.21, *p* = 0.002; high, HR = 2.19, 95% CI 1.64‐2.91, *p* < 0.001).

**DISCUSSION:**

In WRAP, healthier lifestyle in midlife was associated with lower risk of ICI, regardless of *APOE*ε4 carriage or pTau217 level.

**Highlights:**

- Healthier lifestyle is associated with reduced risk of incident impairment.
- Apolipoprotein E (*APOE*) ε4 allele carriage and pTau217 are associated with higher risk of incident impairment.
- No significant lifestyle risk by *APOE*ε4 nor pTau217 interactions were observed.
- Combined lifestyle tertiles and *APOE*ε4 carriage increased risk monotonically.
- Lifestyle tertiles increased impairment risk monotonically across pTau217 groups.

## 1 BACKGROUND

Cognitive impairment and dementia have multifactorial origins, arising from complex, yet incompletely understood interactions between genetic and environmental risk factors. Among these, the apolipoprotein E (*APOE*) ε4 allele is the strongest known genetic risk factor for sporadic Alzheimer's disease (AD)^1^, and a major contributor to cognitive decline. *APOE*ε4 increases dementia risk largely through the amyloid pathway, as evidenced by earlier age at onsets of amyloid accumulation among ε4 carriers.^2^, ^3^ AD is also characterized by a prolonged preclinical phase, during which amyloid and tau pathology — as well as subtle cognitive changes — are detectable years or even decades before a clinical diagnosis of mild cognitive impairment (MCI) or dementia.^4^, ^5^, ^6^, ^7^, ^8^, ^9^ Correspondingly, this extended preclinical period, often beginning in midlife, has become the target window for primary prevention efforts aimed at preserving cognitive health in late‐midlife.

Lifestyle modifications have increasingly been recognized as potential primary prevention strategies for dementia, with growing public and scientific interest in targeting modifiable risk factors.^10^, ^11^, ^12^, ^13^, ^14^, ^15^, ^16^, ^17^ Recent studies^10^, ^18^, ^19^, ^20^ have identified numerous individual lifestyle factors associated with reduced dementia risk. However, relatively few studies have evaluated the combined impact of multiple lifestyle factors,^17^, ^21^, ^22^ and even fewer have assessed these effects in midlife during the preclinical timeframe.^23^, ^24^, ^25^ While there is a prevailing hypothesis suggesting that maintaining a healthy lifestyle may moderate the increased dementia risk associated with *APOE*ε4 carriage, several studies have demonstrated that there is no interaction between *APOE* and lifestyle on dementia risk.^21^, ^26^ As previous studies of lifestyle and *APOE* have focused on late‐life dementia, the interactive effects of lifestyle and *APOE* on midlife cognition remain unresolved. Moreover, the extent to which a healthy lifestyle may moderate cognitive impairment risk related to amyloid pathology remains unknown.

Previously, our group reported no relationship between late‐midlife lifestyle health and amyloid onset, nor a moderating effect of lifestyle on *APOE*ε4‐related or amyloid‐related preclinical cognitive decline.^27^ Building upon this earlier work, we first assessed whether a healthy lifestyle, as indicated by based a weighted composite score of modifiable lifestyle factors (e.g., the “Lifestyle for Brain health” [LIBRA] index^16^, ^28^), reduces risk of incident cognitive impairment (ICI) among middle‐aged, initially unimpaired individuals from the Wisconsin Registry for Alzheimer's Prevention (WRAP). Next, we assessed the impact of midlife lifestyle in the presence of *APOE*ε4 or amyloid burden, as indicated by plasma pTau217‐ALZpath, on risk of ICI. Further, we used a model building approach to probe lifestyle interactions with *APOE*ε4 carriage and amyloid burden to assess whether a healthy midlife lifestyle moderates the negative influence of *APOE*ε4 carriage or amyloid burden on ICI risk.

## 2 METHODS

### 2.1 Participants

Data were from individuals enrolled in WRAP, a longitudinal observational cohort study of initially asymptomatic, middle to late‐middle aged adults enriched for risk of AD based on parental family history.^29^ WRAP participants undergo biennial health and neuropsychological evaluations and have the option to consent to additional biomarker assessments (e.g., blood draw, positron emission tomography (PET), magnetic resonance imaging (MRI), and lumbar puncture). At each study visit, WRAP participants were categorized through a consensus conference review process^30^ as cognitively unimpaired, mild cognitive impairment (MCI), impaired‐not MCI, or dementia; MCI and dementia diagnoses were based on National Institute on Aging–Alzheimer's Association workgroup diagnostic criteria.^31^, ^32^ WRAP participants with complete baseline lifestyle risk factors (defined as their first visit with complete LIBRA risk factor data), genetic data, and two or more visits of cognitive data, who were cognitively unimpaired at baseline, were included in the current study (*n* = 1088; see Figure S1 for CONSORT diagram). *APOE*ε4 was genotyped as previously described.^33^ Participants were categorized as *APOE*ε4 carriers (one or two ε4 alleles) or noncarriers (zero ε4 alleles). All study procedures were approved by the University of Wisconsin‐Madison Institutional Review Board and conducted in accordance with the Helsinki declaration.

**RESEARCH IN CONTEXT**

1. **Systematic review**: The authors reviewed the literature using traditional databases such as PubMed. Lifestyle and genetic risk factors have each been associated with cognitive impairment and dementia risk, and lifestyle modifications are increasingly viewed as possible primary prevention strategies against Alzheimer's disease and dementia. However, few studies have examined the effect of mid‐life lifestyle health on incident cognitive impairment, and the combined influence of lifestyle health and apolipoprotein E (*APOE*) ε4 allele carriage or amyloid on preclinical incident impairment beginning in midlife remains unresolved.
2. **Interpretation**: In the population studied, a healthy lifestyle in midlife was associated with substantially lower risk of incident cognitive impairment, regardless of *APOE*ε4 carriage or pTau217 status.
3. **Future directions**: Results of the current study underscore the importance of lifestyle health in identifying cognitively unimpaired individuals at the greatest risk of impairment and the need for lifestyle and health interventions in midlife.

### 2.2 Neuropsychological assessment and algorithmic definition of impairment

Given the long preclinical phase of AD and the limited number of initially unimpaired participants progressing to clinical impairment (MCI, *n* = 62; dementia, *n* = 18 over the study duration (median(IQR) = 8.2(5.5–10.8) years), we adopted a broader definition of incident cognitive impairment (ICI) to increase power to detect clinically meaningful effect sizes. We used locally‐derived demographically adjusted centiles from a three‐test variant of the Preclinical Alzheimer's Cognitive Composite (PACC‐3), a cognitive composite that has been shown to be sensitive to preclinical Aβ burden as well as an impairment threshold consistent with previous work in WRAP detecting subclinical decline.^9^, ^34^, ^35^, ^36^ The WRAP PACC‐3^37^ is derived from the Rey Auditory Verbal Learning Test total learning score (RAVLT; Trials 1–5),^38^ Wechsler Memory Scale‐Revised (WMS‐R)) Logical Memory delayed recall score,^39^ and the Wechsler Adult Intelligence Scale‐Revised (WAIS‐R) Digit Symbol test score.^40^ Briefly, to obtain the centiles, restricted regression quantiles representing percentiles ranging from tau = 1 to 99 were fit to a set of PACC‐3 scores from cognitively‐healthy individuals.^41^ Cross‐sectional norms estimated these quantiles as a function of age, sex, education, and baseline Wide Range Achievement Test 3rd Edition (WRAT‐3);^42^ longitudinal norms additionally incorporated baseline PACC‐3 score and the number of prior exposures to the test, to account for practice effects. For a given observation, the percentile was estimated as the value of tau for which the error residual was smallest. For each person, ICI was defined as the age at the first visit where PACC‐3 performance fell below the 7th percentile (corresponding to approximately 1.5 SD below the expected mean adjusted for covariates, for a normally distributed outcome), or if never impaired, age at last visit. Primary analyses defined ICI using cross‐sectional norms; secondary analyses used longitudinal norms.

### 2.3 Lifestyle

The LIBRA index was used to assess modifiable lifestyle‐based dementia risk, as it is a validated multivariable lifestyle‐based dementia risk score developed from a systematic review and expert consensus of risk and protective factors for dementia.^14^, ^16^, ^28^ LIBRA is a weighted sum score (theoretical range = ‐5.9 to +12.7; with lower scores indicating healthier lifestyle and lower lifestyle‐based dementia risk) comprised of twelve modifiable factors that can be targeted by lifestyle interventions and primary prevention. Risk factors include physical inactivity, smoking, depression, hypertension, obesity, diabetes, hypercholesterolemia, coronary artery disease, and renal disease. Protective factors include low‐to‐moderate alcohol use, high cognitive activity, and healthy diet. The LIBRA total score was calculated in WRAP (possible range = ‐4.2 to +12.7; Table 1) using a previously reported approach^27^ and following established guidelines^43^, ^44^, ^45^, ^46^, ^47^, ^48^, ^49^, ^50^, ^51^, ^52^, ^53^, ^54^ (detailed description of operationalization in Table 1). Three lifestyle health risk groups were created using baseline LIBRA tertile cutoffs (low risk (i.e., healthiest lifestyle): LIBRA scores between ‐4.2 and 0.1; moderate risk: LIBRA scores between 0.2 to 1.9; high risk (i.e., least healthy lifestyle): LIBRA scores between 2.0 and 7.9).

**TABLE 1:** Operationalization of LIBRA score in WRAP.

LIBRA factor	Definition	Score^a^
Low/moderate alcohol use	Self‐reported frequency of alcohol use. Defined as 2 drinks or less per day for men and 1 drink or less per day for women, including non‐drinkers. Additionally, women reporting a range of 1–2 drinks per day (multi‐choice option) with no further information.^43^	−1.0
Coronary artery disease	Physician review of self‐reported medical history of heart attack (myocardial infarction) or cardiac interventions (e.g., angioplasty, stenting, or coronary bypass surgery).^44^	+1.0
Physical inactivity	Self‐reported physical activity fewer than 150 min of moderate exercise per week (or 7.5 MET hours per week).^45^	+1.1
Renal dysfunction	The CKD‐EPI equation was used to estimate GFR from serum creatinine and other clinical parameters. GFR < 60 mL/min/1.73m^2^; if not available, self‐reported kidney disease.^46^	+1.1
Diabetes	Fasting blood glucose ≥ 126 mg/dL; if not available, self‐reported diabetes.^47^	+1.3
Hypercholesterolemia	Total serum cholesterol ≥ 240 mg/dL; if not available, self‐reported hypercholesterolemia.^48^, ^49^	+1.4
Smoking	Self‐reported history of smoking at least once in the past month.	+1.5
Obesity	BMI ≥ 30 kg/m^2;^ if not available, waist circumference for men > 102 cm and women > 88 cm.^50^	+1.6
Hypertension	Mean systolic blood pressure ≥ 130 mmHg or mean diastolic blood pressure ≥ 80 mmHg; if not available, self‐reported hypertension.^51^	+1.6
Depression	The CES‐D scale was used to measure depressive symptoms. Sum score ≥ 16; if not available, self‐reported depression.^52^, ^53^	+2.1
High cognitive activity	Games frequency of “every day or about every day” on CAS; if not available, Chess, Checkers, or Puzzles frequency of “every day” on FCAS.^54^	−3.2

Abbreviations: BMI, body mass index; CAS, Cognitive Activities Scale; CES‐D, Center for Epidemiologic Studies‐Depression; CKD‐EPI, Chronic Kidney Disease Epidemiology Collaboration; FCAS, Florida Cognitive Activities Scale; GFR, glomerular filtration rate; LIBRA, Lifestyle for BRAin health index; MET, metabolic equivalent.

^a^ LIBRA score components, composed of available factors in the Wisconsin Registry for Alzheimer's Prevention (excluding Mediterranean diet, for which longitudinal data was not available). Negative values assigned to protective factors; positive values assigned to risk factors. Total range ‐5.9 to 12.7 adjusted to ‐4.2 to 12.7 within this study.

### 2.4 ALZpath pTau217 assay

A subset of participants (*n* = 1087) had at least one plasma sample analyzed with the ALZpath Simoa pTau217 assay (Quanterix Part# 104570, Lot# 999008). Samples were analyzed at the UW – Madison ADRC Biomarker Lab on a Quanterix HD‐X in duplicate according to kit instructions.

### 2.5 Statistical analyses

All analyses were performed using R version 4.4.1.^55^ Cox proportional hazards models were fit with the survival^56^, ^57^ package.

Baseline characteristics were compared across LIBRA risk groups and ICI status using Pearson's chi‐squared test or Fisher's exact test for categorical variables, and Wilcoxon rank sum test for continuous variables. Post hoc row‐wise Fisher's exact tests were performed for categorical variables with more than one level and *p *< 0.05.

### 2.6 Incident cognitive impairment and APOEε4

Cox proportional hazard models were used to examine the associations of lifestyle tertiles (i.e., low (reference group), moderate, and high lifestyle‐related risk as indexed by LIBRA) with risk of ICI, in which the respective baseline LIBRA visit was used as the point of entry and right‐censoring occurred if individuals never reached impairment. We used a model building approach to understand the impact of LIBRA on ICI alone and in the presence of *APOE*ε4, where risk of ICI was modeled first with LIBRA alone. Next, risk of ICI was modeled with both LIBRA and *APOE*ε4 carriage (i.e., additive model). Finally, to test whether LIBRA moderates the effect of *APOE*ε4 carriage on risk of ICI, a third model testing their respective interaction was evaluated. The interaction was retained if statistically significant, and the additive and interaction models were compared with likelihood ratio tests (LRT). Finally, to better understand the relative risk associated with different combinations of LIBRA tertiles and *APOE*, we also fit a version of this model that condensed the two main effects and interaction into one six‐level variable encompassing both LIBRA risk group and *APOE*ε4 carrier status, with low lifestyle risk, *APOE*ε4 non‐carriers as the reference. We depicted forest plots of the hazard ratios and confidence intervals across the six groups. For each modeling approach, ICI defined using the cross‐sectional centiles was the primary outcome while ICI defined using longitudinal centiles was the secondary outcome.

### 2.7 ICI and plasma pTau217‐ALZpath

Reference points were derived previously^58^ utilizing the WRAP cohort to define Aβ‐positivity in three pTau217 ranges (low: < 0.40 pg/mL; moderate: 0.40–0.63 pg/mL; high: > 0.63 pg/mL). Few analyzed samples were collected near baseline LIBRA, so the most recent plasma pTau217‐ALZpath measure was chosen for each participant (*n* = 1087; mean(SD) time from baseline LIBRA to pTau217 = 10.7(3.2) years). In line with our *APOE* analyses, we followed a parallel process of model building to evaluate the relationship between LIBRA and risk of ICI alone and with three pTau217 levels, as well as the interaction between these factors. To better understand the relative risk associated with different combinations of LIBRA and pTau217 ranges, the low lifestyle risk, pTau217 low range was used as the reference for a nine‐level variable combining LIBRA risk groups and pTau217. Primary and secondary outcomes were as above.

### 2.8 Sensitivity analyses

To evaluate the dependence of our results on model parameterization, we conducted several sets of sensitivity analyses. First, we examined whether our results were sensitive to our choice of cutpoints for LIBRA by predicting risk of ICI using the continuous LIBRA score instead of LIBRA tertiles, for both primary and secondary outcomes separately. Second, because we have previously observed a high rate of reversion (∼50%)^30^ within those classified as impaired by consensus conference, we repeated all models after applying more conservative definitions of ICI requiring two consecutive visits below the 7th percentile, again for both primary and secondary outcomes separately. Lastly, we repeated these more conservative analyses once more, again using the continuous LIBRA score instead of LIBRA tertiles.

The proportional hazards assumption for all Cox regressions was assessed through the interaction between time and the Schoenfeld residuals.^59^ Harrell concordance rates (C‐statistic(s)) for censored data were calculated as a measure of predictive accuracy.^60^ Statistical significance was set at *p *< 0.05.

## 3 RESULTS

### 3.1 Sample characteristics

Baseline sample characteristics and frequency of lifestyle factors in WRAP are presented overall and by incident cognitive impairment (Table 2). Over a median (IQR) of 8.2 (5.5–10.8) years of cognitive follow‐up, 278 (25.6%) participants were classified with ICI based on demographically‐adjusted internal PACC‐3 cross‐sectional norms. Those who became impaired over the study duration were significantly older at baseline than their unimpaired counterparts (mean difference (95% CI) = ‐1.8 (‐2.7, ‐0.8),[Fig alz70573-fig-0001] *p* < 0.001). A greater proportion of non‐Hispanic/Latino White participants remained unimpaired (*X* ^2^ (1, *N* = 1039) = 7.15, *p* = 0.004), whereas those who identify as Black/African American were more frequently categorized as impaired using this approach (*X* ^2^(1, *N* = 28) = 16.83, *p* < 0.001). Physical inactivity, smoking, and depression were more common risk factors in the ICI group, and high cognitive activity was a more common protective factor in the unimpaired group. Additionally, recent consensus conference cognitive diagnoses significantly differed, where the ICI group was more often diagnosed with MCI, non‐MCI cognitive impairment, and dementia. In the plasma pTau217‐ALZpath subset, those unimpaired were significantly younger at time of pTau217 and had fewer years between time of event (i.e., latest visit) and time of pTau217.

**TABLE 2:** Sample characteristics and LIBRA risk factors by incident cognitive impairment as defined by cross‐sectional norms.

Variable	Overall, *N* = 1088	Impaired^a^, *N* (%) = 278 (25.6%)	Unimpaired^a^, *N* (%) = 810 (74.4%)	*p*‐value^b^	Row‐wise *p*‐value^b^
Age at baseline LIBRA	57.8 (6.5)	59.1 (6.1)	57.4 (6.6)	<0.001	
No. study visits, median (IQR)	4 (3–5)	4 (3–5)	4 (3–5)	0.10	
Years of cognitive follow‐up, median (IQR)	8.2 (5.5–10.8)	8.0 (5.3–10.4)	8.3 (5.7–10.9)	0.20	
Most recent clinical diagnosis				<0.001	
CU	953 (92%)	191 (73%)	762 (98%)		<0.001
MCI	62 (6.0%)	50 (19%)	12 (1.5%)		<0.001
Impaired, not MCI	8 (0.8%)	5 (1.9%)	3 (0.4%)		0.03
Dementia	18 (1.7%)	15 (5.7%)	3 (0.4%)		<0.001
*APOE*ε4 carriers	416 (38%)	136 (49%)	280 (35%)	<0.001	
Female	757 (70%)	206 (74%)	551 (68%)	0.06	
Non‐Hispanic, White^c^	1,039 (95%)	257 (92%)	782 (97%)	0.004	
Black/African American^c^	28 (2.6%)	17 (6.1%)	11 (1.4%)	<0.001	
Years of education, median (IQR)	16 (14–18)	16 (14–18)	16 (14–18)	0.60	
WRAT‐3, median (IQR)	107 (100–113)	107 (100–113)	107 (100–113)	0.50	
ICI cross‐sectional PACC‐3 z‐score^a^, median (IQR)	0.42 (0.07–0.75)	0.03 (0.02–0.05)	0.58 (0.24–0.84)	<0.001	
Baseline LIBRA score^d^, median (IQR)	0.6 (‐1.0–2.2)	1.4 (0.1–3.2)	0.6 (‐1.0–2.2)	<0.001	
Baseline LIBRA tertile^d^				<0.001	
Low	442 (41%)	83 (30%)	359 (44%)		<0.001
Moderate	307 (28%)	85 (31%)	222 (27%)		0.32
High	339 (31%)	110 (40%)	229 (28%)		<0.001
Baseline LIBRA factors^d^					
Low/moderate alcohol use	933 (86%)	242 (87%)	691 (85%)	0.50	
High cognitive activity	225 (21%)	37 (13%)	188 (23%)	<0.001	
Coronary artery disease	23 (2.1%)	8 (2.9%)	15 (1.9%)	0.30	
Physical inactivity	346 (32%)	104 (37%)	242 (30%)	0.02	
Renal dysfunction	51 (4.7%)	17 (6.1%)	34 (4.2%)	0.20	
Diabetes	49 (4.5%)	18 (6.5%)	31 (3.8%)	0.07	
Hypercholesterolemia	169 (16%)	49 (18%)	120 (15%)	0.30	
Smoking	63 (5.8%)	26 (9.4%)	37 (4.6%)	0.003	
Obesity	393 (36%)	108 (39%)	285 (35%)	0.30	
Hypertension	487 (45%)	131 (47%)	356 (44%)	0.40	
Depression	138 (13%)	51 (18%)	87 (11%)	0.001	
Plasma pTau217‐ALZpath subset	1087	278	809	–	
Age at latest pTau217	68.6 (6.9)	69.6 (6.4)	68.2 (7.1)	0.004	
Time from ICI to latest pTau217	1.0 (3.2)	4.3 (4.0)	−0.2 (1.7)	<0.001	
Latest pTau217 range^e^				<0.001	
pTau217 Low	651 (60%)	139 (50%)	512 (63%)		<0.001
pTau217 Moderate	227 (21%)	52 (19%)	175 (22%)		0.35
pTau217 High	209 (19%)	87 (31%)	122 (15%)		<0.001

*Note*: Values are mean (SD) or No. (%), unless otherwise noted.

Abbreviations: APOE, apolipoprotein E; BMI, body mass index; CU, cognitively unimpaired; ICI, incident cognitive impairment; IQR, interquartile range; LIBRA, Lifestyle for BRAin health index; MCI, mild cognitive impairment; PACC‐3, Preclinical Alzheimer's Cognitive Composite (three‐test version with Digit Symbol); WRAT‐3, Wide Range Achievement Test (3rd edition).

^a^ Incident cognitive impairment definition using cross‐sectional PACC‐3 norms. Impairment defined as first impaired score (score below 0.07), or most recent score if never impaired.

^b^ Statistical tests: *χ* ^2^ or Fisher's exact test for categorical variables, Wilcoxon rank sum test for continuous variables. Post hoc row‐wise Fisher's exact test comparing each row versus the sum of the others for categorical variables with more than one level and *p* < 0.05.

^c^ Participants can report multiple races. Representative of reporting instances.

^d^ Representative of baseline LIBRA score (defined as first full LIBRA score). Low risk (‐4.2–0.1), moderate risk (0.2–1.9), high risk (2.0–7.9).

^e^ Plasma pTau217‐ALZpath cut‐offs at 0.40 pg/mL and 0.63 pg/mL.

Table S1 displays baseline characteristics stratified by the secondary definition of ICI (as defined by longitudinal norms), under which 304 (27.9%) participants were classified as impaired. Briefly, impaired participants were marginally older and had greater years of cognitive follow‐up. Differences by impairment for self‐reported race and LIBRA risk factors were not observed. Cognitive diagnoses continued to differ by ICI group, except for non‐MCI impairment. Group differences within the pTau217‐ALZpath subset were qualitatively similar to the primary analysis. Sample characteristics by baseline LIBRA tertile are shown in Table S2. Notably, the low‐risk group had higher PACC‐3 *z*‐scores and was comprised of more females than the moderate and high‐risk groups. Individuals in the high‐risk group had lower WRAT‐3 scores and years of education compared to both the low and moderate risk groups. The proportion of individuals at risk within each LIBRA factor increased stepwise across the LIBRA tertiles (i.e., low < moderate < high risk).[Fig alz70573-fig-0002]

### 3.2 Risk of incident impairment and lifestyle

**FIGURE 1 alz70573-fig-0001:**
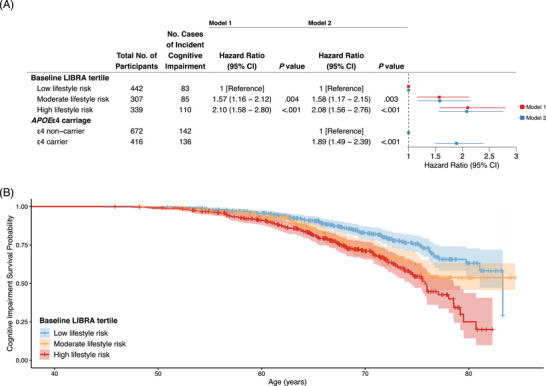
Cox proportional hazard model output (A) and survival curves (B) based on ICI as defined by PACC‐3 performance below 7th percentile on cross‐sectional internal PACC‐3 norms (adjusted for age, sex, education, and WRAT‐3). Model 1: baseline LIBRA risk tertile; Model 2: baseline LIBRA risk tertile and *APOE*ε4 carriage. *APOE*, apolipoprotein E; ICI, incident cognitive impairment; LIBRA, Lifestyle for Brain health index; PACC‐3, Preclinical Alzheimer's Cognitive Composite (three‐test version with Digit Symbol); WRAT‐3, Wide Range Achievement Test 3rd Edition.

In analyses of the primary outcome (ICI defined using cross‐sectional norms), participants with high or moderate lifestyle risk (i.e., least healthy lifestyles) at baseline had significantly greater risk for developing ICI compared to participants with low lifestyle risk (i.e., most healthy lifestyle; moderate: HR, 1.57 [95% CI, 1.16–2.12], *p* = 0.004; high: HR, 2.10 [95% CI, 1.58–2.80], *p* < 0.001; see Figure 1 for results and Table S3 for full model output). Analysis of the secondary outcome (ICI defined using longitudinal norms; *n* = 304 (27.9%) impaired) no longer showed a difference between moderate and low lifestyle risk, and only a marginally significant increase in risk of ICI for those with high lifestyle risk (Figure S2 and Table S3).

Moreover, sensitivity analyses indicated that these results were fairly robust to different definitions of ICI and lifestyle risk. In particular, the group with less healthy lifestyles had greater risk for ICI than the healthy‐lifestyle group whether LIBRA was parameterized categorically or continuously, and whether one or two consecutive low‐centile PACC‐3 scores were required for ICI. Analogous to the secondary outcome, under all the sensitivity definitions of ICI, the moderate and low lifestyle risk groups no longer differed (Tables S3 and S4 and Figures S3 and S4).

### 3.3 Risk of incident impairment according to lifestyle and APOEε4

**FIGURE 2 alz70573-fig-0002:**
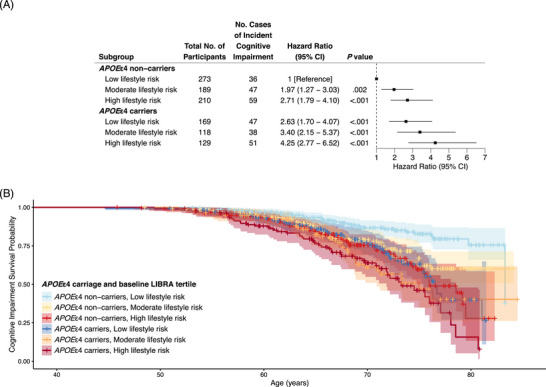
Cox proportional hazard model output (A) and survival curves (B), based on ICI as defined by PACC‐3 performance below 7th percentile on cross‐sectional internal PACC‐3 norms (adjusted for age, sex, education, and WRAT‐3), for a six‐level combination of baseline LIBRA tertile and *APOE*ε4 carriage (6 categories with low lifestyle risk, *APOE*ε4 non‐carriers as the reference). *APOE*, apolipoprotein E; ICI, incident cognitive impairment; LIBRA, Lifestyle for Brain health index; PACC‐3, Preclinical Alzheimer's Cognitive Composite (three‐test version with Digit Symbol); WRAT‐3, Wide Range Achievement Test 3rd Edition.

**FIGURE 3:**
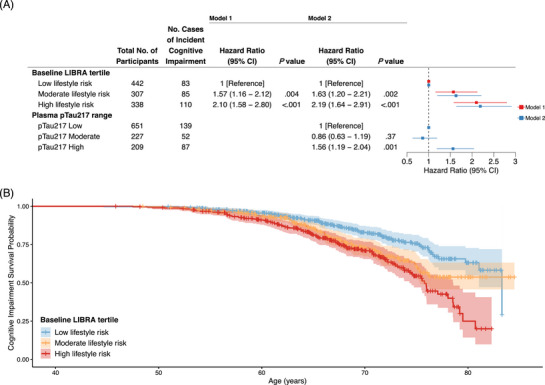
Cox proportional hazard model output (A) and survival curves (B) based on ICI as defined by PACC‐3 performance below 7th percentile on cross‐sectional internal PACC‐3 norms (adjusted for age, sex, education, and WRAT‐3). Model 1: baseline LIBRA risk tertile; Model 2: baseline LIBRA risk tertile and plasma pTau217‐ALZpath range. *APOE*, apolipoprotein E; ICI, incident cognitive impairment; LIBRA, Lifestyle for Brain health index; PACC‐3, Preclinical Alzheimer's Cognitive Composite (three‐test version with Digit Symbol); WRAT‐3, Wide Range Achievement Test 3rd Edition.

**FIGURE 4:**
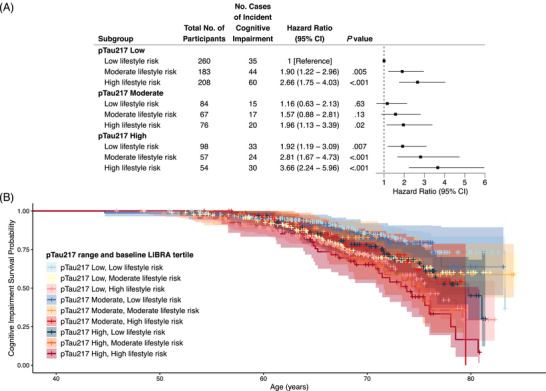
Cox proportional hazard model output (A) and survival curves (B), based on ICI as defined by PACC‐3 performance below 7th percentile on cross‐sectional internal PACC‐3 norms (adjusted for age, sex, education, and WRAT‐3), for a nine‐level combination of baseline LIBRA tertile and plasma pTau217‐ALZpath range (9 categories with low lifestyle risk, pTau217 low as the reference). *APOE*, apolipoprotein E; ICI, incident cognitive impairment; LIBRA, Lifestyle for Brain health index; PACC‐3, Preclinical Alzheimer's Cognitive Composite (three‐test version with Digit Symbol); WRAT‐3, Wide Range Achievement Test 3rd Edition.

After adjusting for *APOE*ε4 carriage, estimated effects of lifestyle risk on ICI remained similar across all definitions (moderate: HR, 1.58 [95% CI, 1.17–2.15], *p* = 0.003; high: HR, 2.08 [95% CI, 1.56–2.76], *p* < 0.001; Figure 1A, Figures 2A–4A, Tables S3 and S4). No significant interactions between baseline LIBRA and *APOE*ε4 carriage were observed in relation to risk of ICI (Tables S3 and S4), indicating that the association of lifestyle with ICI did not vary substantially by *APOE*ε4 carriage. LRTs confirmed interaction terms did not improve model fit. When observed alone, *APOE*ε4 carriers were 1.89 (95% CI, 1.49–2.39, *p* < 0.001) times more likely to develop ICI compared to *APOE*ε4 non‐carriers (Figure 1A). Combining *APOE*ε4 carriage and LIBRA tertiles into a six‐level variable produced a monotonic association with an increasingly unhealthy lifestyle (high lifestyle risk) and increasing genetic risk (*APOE*ε4 carriers; Figure 2). *APOE*ε4 carriers with high lifestyle risk were 4.25 (95% CI, 2.77–6.52, *p* < 0.001) times more likely to develop ICI compared to *APOE*ε4 non‐carriers with low lifestyle risk over the study duration (see Figure 2 for results and Table S5 for full model output). Secondary and sensitivity definitions of ICI followed similar associations with lifestyle risk and *APOE*ε4 carriage but failed to exhibit a difference between the low and moderate lifestyle risk groups within *APOE*ε4 non‐carriers (Figures S5–S7 and Table S5).

### 3.4 Risk of incident impairment according to lifestyle and plasma pTau217‐ALZpath

All but one participant had an available pTau217‐ALZpath measure (*n* = 1087). In models accounting for pTau217 range, the estimated effects of lifestyle risk on ICI remained similar across all definitions (moderate: HR, 1.63 [95% CI, 1.20–2.21], *p* = 0.002; high: HR, 2.19 [95% CI, 1.64–2.91], *p* < 0.001; Figure 3A, Tables S6 and S7, Figures S8A–S10A). A significant main effect of pTau217 was observed, in which those with high pTau217 measures were 1.56 (95% CI, 1.19–2.04, *p* = 0.001) times more likely to develop ICI compared to those with low pTau217, but moderate pTau217 measures did not differ from low measures (Figure 3A). This pattern of significance was observed across all definitions of ICI (Tables S6 and S7). No significant lifestyle and pTau217 interactions were observed when defining lifestyle risk in tertiles and LRTs proved no difference in model fit. However, a significant interaction was observed within one sensitivity definition of ICI when using continuous LIBRA, but this interaction also failed to significantly improve model fit (𝜒^2^(2) = 5.38, *p* = 0.07). Table S7 depicts the significant interaction between continuous LIBRA and high pTau217 under the ICI definition of two consecutive low‐centile PACC‐3 scores on cross‐sectional norms (HR, 0.81 [95% CI, 0.68–0.99], *p* = 0.03).

The nine‐level variable combining LIBRA tertiles and pTau217 ranges extends the results from the model including each as main effects. As Figure 4 shows, within each pTau217 range, we saw monotonic associations of increasingly unhealthy lifestyle. Consistent with the main effects model, individuals with high pTau217 measures and high lifestyle risk had the highest HR relative to the low pTau217, low lifestyle risk reference group. Specifically, people with high lifestyle risk and high pTau217 were 3.66 (95% CI, 2.24–5.96, *p* < 0.001) times more likely to develop ICI compared to those with low pTau217 measures and low lifestyle risk (see Figure 4 for results and Table S8 for full model output). Using sensitivity definitions of ICI, HR patterns of significance and magnitude were largely consistent with the exception of low pTau217, moderate lifestyle risk and moderate pTau217, high lifestyle risk no longer differing from 0 in sensitivity analyses (Table S8 and Figures S11–S13).

## 4 DISCUSSION

In this initially cognitively unimpaired, middle to late‐middle‐aged cohort, we explored the impact of modifiable lifestyle health alone and in the presence of *APOE*ε4 carriage and pTau217 levels on risk of ICI over a median of 8.2 years of follow‐up. While a healthy lifestyle (i.e., a low LIBRA score) in midlife was consistently associated with lower risk of ICI both in the presence of *APOE*ε4 carriage and in the presence of elevated pTau217, LIBRA did not moderate the negative impact of *APOE*ε4 or pTau217 on risk of ICI. Notably, these associations remained largely consistent across multiple definitions of preclinical impairment. However, the absence of a differential finding by *APOE*ε4 carriage or by pTau217 protein concentration suggests that the salutary effect of lifestyle is rather general instead of oppositional to the AD pathway specifically. Collectively, these results underscore both the importance of lifestyle health in identifying cognitively unimpaired individuals at the greatest risk of future impairment and the possibility of identifying useful lifestyle and health interventions in midlife.

This study is among the first to evaluate a comprehensive, weighted lifestyle index in relation to preclinical cognitive impairment within a well‐characterized initially unimpaired cohort, offering novel insights into the preclinical phase of AD. For example, compared to several population and patient‐based cohort studies have shown that LIBRA predicts MCI and dementia,^24^, ^28^, ^61^, ^62^ the present results add to recent cross‐sectional findings^63^ and suggest that LIBRA may also have predictive utility prior to the emergence of clinically significant symptoms. Differentiating disease‐related cognitive change from the more gradual age‐related cognitive decline is particularly challenging in the early, pre‐symptomatic phase of AD. To detect subtle impairment in WRAP, we considered several definitions of ICI based on demographically‐adjusted PACC‐3 norms, including abnormal cognitive performance relative to peers (cross‐sectional norms), abnormal cognitive performance relative to an individual's past performance (longitudinal norms), and two consecutive visits of abnormal cognitive performance on either norm. A lower LIBRA (i.e., healthier lifestyle) was consistently associated with lower risk of ICI compared to a higher LIBRA across all definitions of impairment. In a previous paper in this cohort,^27^ which modeled continuous cognitive outcomes rather than impairment, we observed an overall main effect of LIBRA on cognition, but no interaction with age. Taken together, one possible interpretation of our group's findings is that a healthy lifestyle supports a better baseline of cognitive performance, which then provides “breathing room” before a given quantity of decline hits a threshold for impairment. These results underline the importance of a healthy lifestyle on the preservation of cognitive functioning and emphasize the potential utility of LIBRA as a useful tool for identifying individuals at increased risk of impairment in midlife.

In WRAP, a healthy lifestyle was associated with lower risk of ICI in both *APOE*ε4 carriers and non‐carriers. We found that, in general, *APOE*ε4 carriers had significantly higher risk of ICI compared to *APOE*ε4 non‐carriers and showed further that *APOE*ε4 carriers with an unhealthy lifestyle had significantly higher risk of ICI compared to *APOE*ε4 non‐carriers with a healthy lifestyle. Consistent with a recent study of LIBRA and dementia risk,^26^, ^61^ the data did not indicate a significant interaction between *APOE*ε4 carriage and LIBRA with risk of impairment. Notably, these results are also in agreement with a previous study^21^ of 196,383 initially unimpaired individuals in the UK Biobank, where a healthy lifestyle was associated with lower risk of dementia across individuals with high and low levels of AD genetic risk. Taken together, these findings suggest that lifestyle health is consistently associated with reduced risk of cognitive impairment regardless of *APOE*ε4 carriage and spanning subtle preclinical cognitive impairment in mid‐ to late‐life dementia.

Similarly, in WRAP, a healthy lifestyle was associated with lower risk of ICI across all levels of pTau217, supporting the notion that lifestyle health benefits cognitive outcomes regardless of underlying amyloid burden. However, consistent with our *APOE* findings, we observed no statistical interaction between pTau217 and LIBRA, indicating that the increased risk of ICI associated with amyloid pathology is not moderated by lifestyle health. These results are consistent with recent studies^22^, ^64^, ^65^ suggesting that, while lifestyle factors may not modify risk of amyloid‐related cognitive impairment, they do provide general protective effects on cognitive health.

This builds on our prior work in WRAP, which found no association between lifestyle and amyloid PET accumulation.^27^, ^66^, ^67^ Importantly, these results also reinforce that even among those at high genetic or biomarker risk of AD, a healthy lifestyle was associated with reduced ICI risk compared to an unhealthy lifestyle. This supports the value of promoting healthy lifestyle behaviors broadly as a public health strategy, independent of genetic or biomarker‐risk profiles.

Perhaps counterintuitively, this conclusion may have new significance in the era of disease‐modifying therapies. It is estimated that 66% of amyloid‐positive individuals have at least one ε4 allele,^68^ and approved anti‐amyloid therapies such as lecanemab have been observed to have worse risk‐benefit ratios in ε4 homozygotes, with no significant improvement on the cognitive endpoint and increased risk of adverse events.^69^, ^70^, ^71^ Our results suggest that maintaining a healthy lifestyle in midlife may reduce the risk of cognitive impairment among *APOE*ε4 carriers, highlighting the continued importance of primary prevention efforts – even in the context of emerging disease modifying treatments.

The potential of multidomain cardiovascular and lifestyle interventions to reduce cognitive decline and risk of dementia has been evaluated in several clinical trials. While no significant reduction in all‐cause dementia risk or cognitive decline was observed in the Prevention of Dementia by Intensive Vascular care (PreDIVA)^72^ or Multidomain Alzheimer Preventive Trial (MAPT),^73^ the multidomain lifestyle intervention strategy as implemented in the Finnish Geriatric Intervention Study to Prevent Cognitive Impairment and Disability (FINGER)^18^ trial reported modest cognitive benefits from a multidomain lifestyle intervention. Notably, this effect was independent of the baseline LIBRA score,^74^ and the FINGER intervention has recently been shown to be a potentially cost‐effective dementia prevention strategy.^75^ However, the absence of comparable findings examining the effect of early lifestyle interventions on long‐term cognitive outcomes in midlife underscores a critical gap. A life course approach is increasingly recognized as essential to understanding how modifiable lifestyle factors contribute to cognitive outcomes throughout life. Ongoing efforts such as the U.S.‐based POINTER study aim to replicate FINGER, but more research is needed to examine whether lifestyle interventions in midlife can meaningfully impact risk of future cognitive impairment.^76^, ^77^

### 4.1 Limitations

The current study has several limitations. We did not consider ε4 count, only ε4 carriage, so we could not assess potential differences in the effect of *APOE*ε4 hetero‐ versus homozygosity on the association of LIBRA with ICI. Further, to make the most of available data, we used the last‐available pTau217 measure rather than the baseline pTau217 measure. Although, we can assume amyloid accumulation has occurred between the time of baseline LIBRA and pTau217 due to the slow progression of AD. Additionally, unimpaired participants were significantly younger at time of pTau217 measure than their impaired counterparts. Participants with better lifestyle health may have been more likely to participate in continued serial testing, and participants had a relatively healthy lifestyle overall, which may have led to underrepresentation of those with higher lifestyle‐based dementia risk. As the WRAP study primarily includes participants from the upper Midwest population, and this specific sample largely included non‐Hispanic/Latino White participants (95%), the generalizability of results may be limited. Further, given that WRAP is enriched for AD risk due to parental family history, this poses a selection bias that may further limit generalizability.

### 4.2 Conclusions

A healthy lifestyle, as measured by the LIBRA index, is associated with reduced risk of incident preclinical cognitive impairment among middle aged individuals in WRAP, and this risk reduction remains in the presence of *APOE*ε4 or elevated pTau217. As the cohort progresses toward a clinical endpoint of MCI or dementia, future work focused on clinical disease progression will help to solidify our understanding of the impact of lifestyle health across the preclinical and clinical AD spectrum.

## CONFLICT OF INTEREST STATEMENT

E.M.J. serves on a data monitoring committee for an NIA award, and her spouse is employed by and has stock in Epic Systems Corporation, a biomedical company. S.C.J. has participated on advisory panels for ALZpath and Enigma Biomedical. Authors R.L.S., K.A.C., J.E.O., N.A.C., K.D.M., B.P.H., and R.E.L. declare they have no competing interests. Author disclosures are available in the Supporting Information.

## CONSENT STATEMENT

All participants provided informed consent.

## Supporting information



Supporting Information

Supporting Information
