# Supplementary material for: Lifestyle, genetic risk, plasma pTau217, and incident cognitive impairment in WRAP
Source: Alzheimers Dement. 2025 Aug 13;21(8):e70573. doi: 10.1002/alz.70573 (PMC12344448; doi:10.1002/alz.70573)
Supplement: Supplementary file 2 — Supporting Information [file ALZ-21-e70573-s002.docx]

**Supplementary Table 1. Sample characteristics and LIBRA risk factors by incident cognitive impairment as defined by longitudinal norms.**

| **Variable** | **Overall,**  N = 1088 | **Impaired^*^,**  N (%) = 304 (27.9%) | **Unimpaired^*^,**  N (%) = 784 (72.1%) | ***P* value^†^** | **Row-wise *P* value^†^** |
| --- | --- | --- | --- | --- | --- |
| **Age at baseline LIBRA** | 57.8 (6.5) | 58.5 (6.5) | 57.6 (6.5) | .05 |  |
| **No. study visits, Median (IQR)** | 4 (3 - 5) | 4 (3 - 5) | 4 (3 - 5) | .002 |  |
| **Years of cognitive follow-up, Median (IQR)** | 8.2 (5.5 - 10.8) | 8.8 (6.5 - 11.5) | 7.9 (5.3 - 10.4) | <.001 |  |
| **Most recent**  **clinical diagnosis** |  |  |  | <.001 |  |
| *CU* | 953 (92%) | 225 (78%) | 728 (97%) |  | <.001 |
| *MCI* | 62 (6.0%) | 45 (16%) | 17 (2.3%) |  | <.001 |
| *Impaired, not MCI* | 8 (0.8%) | 2 (0.7%) | 6 (0.8%) |  | >.90 |
| *Dementia* | 18 (1.7%) | 17 (5.9%) | 1 (0.1%) |  | <.001 |
| ***APOE*ε4 carriers** | 416 (38%) | 142 (47%) | 274 (35%) | <.001 |  |
| **Female** | 757 (70%) | 221 (73%) | 536 (68%) | .20 |  |
| **Non-Hispanic/Latino, White^‡^** | 1,039 (95%) | 287 (94%) | 752 (96%) | .30 |  |
| **Black/African American^‡^** | 28 (2.6%) | 10 (3.3%) | 18 (2.3%) | .40 |  |
| **Years of education, Median (IQR)** | 16 (14 - 18) | 16 (14 - 18) | 16 (14 - 18) | .80 |  |
| **WRAT-3,**  **Median (IQR)** | 107 (100 - 113) | 107 (101 - 113) | 107 (99 - 113) | .20 |  |
| **ICI longitudinal** **PACC-3 z-score^*^, Median (IQR)** | 0.31 (0.06 - 0.63) | 0.03 (0.02 - 0.06) | 0.48 (0.26 - 0.74) | <.001 |  |
| **Baseline LIBRA score**^§^**, Median (IQR)** | 0.6 (-1.0 - 2.2) | 0.6 (-1.0 - 2.7) | 0.6 (-1.0 - 2.2) | .12 |  |
| **Baseline LIBRA tertile**^§^ |  |  |  | .60 |  |
| *Low* | 442 (41%) | 116 (38%) | 326 (42%) |  |  |
| *Moderate* | 307 (28%) | 88 (29%) | 219 (28%) |  |  |
| *High* | 339 (31%) | 100 (33%) | 239 (30%) |  |  |
| **Baseline LIBRA factors^‡^** |  |  |  |  |  |
| **Low/moderate alcohol use** | 933 (86%) | 264(87%) | 669 (85%) | .50 |  |
| **High Cognitive Activity** | 225 (21%) | 58 (19%) | 167 (21%) | .40 |  |
| **Coronary artery disease** | 23 (2.1%) | 8 (2.6%) | 15 (1.9%) | .50 |  |
| **Physical inactivity** | 346 (32%) | 106 (35%) | 240 (31%) | .20 |  |
| **Renal dysfunction** | 51 (4.7%) | 15 (4.9%) | 36 (4.6%) | .80 |  |
| **Diabetes** | 49 (4.5%) | 15 (4.9%) | 34 (4.3%) | .70 |  |
| **Hypercholesterolemia** | 169 (16%) | 47 (15%) | 122 (16%) | >.90 |  |
| **Smoking** | 63 (5.8%) | 22 (7.2%) | 41 (5.2%) | .20 |  |
| **Obesity** | 393 (36%) | 113 (37%) | 280 (36%) | .70 |  |
| **Hypertension** | 487 (45%) | 137 (45%) | 350 (45%) | .90 |  |
| **Depression** | 138 (13%) | 47 (15%) | 91 (12%) | .09 |  |
| **Plasma pTau217-ALZpath subset** | 1087 | 304 | 783 | - |  |
| **Age at latest pTau217** | 68.6 (6.9) | 69.6 (6.7) | 68.2 (7.0) | .003 |  |
| **Time from ICI to latest pTau217** | 1.0 (3.2) | 2.3 (3.9) | 0.5 (2.7) | <.001 |  |
| **Latest pTau217 range**^¶^ |  |  |  | <.001 |  |
| *pTau217 Low* | 651 (60%) | 150 (49%) | 501 (64%) |  | <.001 |
| *pTau217 Moderate* | 227 (21%) | 57 (19%) | 170 (22%) |  | .32 |
| *pTau217 High* | 209 (19%) | 97 (32%) | 112 (14%) |  | <.001 |
| Values are Mean (SD) or No. (%), unless otherwise noted.  ^*^Incident cognitive impairment definition using longitudinal PACC-3 norms. Impairment defined as first impaired score (score below 0.07), or most recent score if never impaired.  ^†^Statistical tests: χ^2^ or Fisher's exact test for categorical variables, Wilcoxon rank sum test for continuous variables. Post hoc row-wise Fisher’s exact test comparing each row versus the sum of the others for categorical variables with more than one level and *P* <.05.  **^‡^**Participants can report multiple races. Representative of reporting instances.  ^§^Representative of baseline LIBRA score (defined as first full LIBRA score).  ^¶^Plasma pTau217-ALZpath cut-offs at 0.40 pg/mL and 0.63 pg/mL.  Abbreviations: LIBRA, Lifestyle for BRAin health index; APOE, apolipoprotein E; CU, cognitively unimpaired; MCI, mild cognitive impairment; WRAT-3, Wide Range Achievement Test (3rd edition); PACC-3, Preclinical Alzheimer's Cognitive Composite (three-test version with Digit Symbol). | | | | | |

**Supplementary Table 2: Sample characteristics and LIBRA risk factors by baseline LIBRA tertile.**

| **Variable** | **Overall,**  N = 1088 | **1. Low Risk^*^,**  N (%) = 442 (%) | **2. Moderate Risk^*^,**  N (%) = 307 (%) | **3. High Risk^*^,**  N (%) = 339 (%) | **Group *P* value^†^** | **Pairwise differences^†^** |
| --- | --- | --- | --- | --- | --- | --- |
| **Age at baseline LIBRA** | 57.8 (6.5) | 58.2 (6.4) | 57.9 (6.7) | 57.3 (6.4) | .20 |  |
| **No. study visits, Median (IQR)** | 4 (3 - 5) | 4.0 (3 - 5) | 4.0 (3 - 5) | 4.0 (3 - 5) | .002 | 3 vs. 1, 2 |
| **Years of cognitive follow-up, Median (IQR)** | 8.2 (5.5 - 10.8) | 8.6 (6.1 - 11.1) | 8.1 (5.9 - 10.3) | 7.6 (4.8 - 10.0) | .001 | 3 vs. 1, 2 |
| **Most recent**  **clinical diagnosis** |  |  |  |  | .10 |  |
| *CU* | 953 (92%) | 394 (92%) | 270 (92%) | 289 (90%) |  |  |
| *MCI* | 62 (6.0%) | 21 (4.9%) | 17 (5.8%) | 24 (7.5%) |  |  |
| *Impaired, not MCI* | 8 (0.8%) | 1 (0.2%) | 2 (0.7%) | 5 (1.6%) |  |  |
| *Dementia* | 18 (1.7%) | 12 (2.8%) | 3 (1.0%) | 3 (0.9%) |  |  |
| ***APOE*ε4 carriers** | 416 (38%) | 169 (38%) | 118 (38%) | 129 (38%) | >0.9 |  |
| **Female** | 757 (70%) | 332 (75%) | 199 (65%) | 226 (67%) | .004 | 1 vs. 2, 3 |
| **Non-Hispanic/Latino, White^‡^** | 1,039 (95%) | 424 (96%) | 295 (96%) | 320 (94%) | .50 |  |
| **Black/African American^‡^** | 28 (2.6%) | 4 (0.9%) | 7 (2.3%) | 17 (5.0%) | .001 | 1 vs. 3 |
| **Years of education, Median (IQR)** | 16 (14 - 18) | 16 (14 - 18) | 16 (14 - 18) | 16 (14 - 17) | <.001 | 3 vs. 1, 2 |
| **WRAT-3,**  **Median (IQR)** | 107 (100 - 113) | 109.0 (103 - 113) | 108.0 (101 - 113) | 105.0 (98 - 111) | <.001 | 3 vs. 1, 2 |
| **ICI cross-sectional** **PACC-3 z-score^*^, Median (IQR)** | 0.42 (0.07 - 0.75) | 0.49 (0.18 - 0.82) | 0.39 (0.07 - 0.70) | 0.35 (0.06 - 0.70) | <.001 | 1 vs. 2, 3 |
| **Baseline LIBRA factors^*^** |  |  |  |  |  |  |
| **Low/moderate alcohol use** | 933 (86%) | 401 (91%) | 274 (89%) | 258 (76%) | <.001 | 3 vs. 1, 2 |
| **High Cognitive Activity** | 225 (21%) | 190 (43%) | 25 (8.1%) | 10 (2.9%) | <.001 | All Pairs |
| **Coronary artery disease** | 23 (2.1%) | 5 (1.1%) | 7 (2.3%) | 11 (3.2%) | .12 |  |
| **Physical inactivity** | 346 (32%) | 98 (22%) | 83 (27%) | 165 (49%) | <.001 | 3 vs. 1, 2 |
| **Renal dysfunction** | 51 (4.7%) | 10 (2.3%) | 15 (4.9%) | 26 (7.7%) | .002 | 1 vs. 3 |
| **Diabetes** | 49 (4.5%) | 1 (0.2%) | 15 (4.9%) | 33 (9.7%) | <.001 | All pairs |
| **Hypercholesterolemia** | 169 (16%) | 31 (7.0%) | 46 (15%) | 92 (27%) | <.001 | All pairs |
| **Smoking** | 63 (5.8%) | 7 (1.6%) | 16 (5.2%) | 40 (12%) | <.001 | All pairs |
| **Obesity** | 393 (36%) | 66 (15%) | 94 (31%) | 233 (69%) | <.001 | All pairs |
| **Hypertension** | 487 (45%) | 83 (19%) | 148 (48%) | 256 (76%) | <.001 | All pairs |
| **Depression** | 138 (13%) | 9 (2.0%) | 27 (8.8%) | 102 (30%) | <.001 | All pairs |
| **Plasma pTau217-ALZpath subset** | 1087 | 442 | 307 | 338 | .60 |  |
| **Age at latest pTau217** | 68.6 (6.9) | 69.0 (7.0) | 68.8 (6.8) | 67.8 (6.9) | .07 |  |
| **Time from baseline LIBRA to latest pTau217** | 10.7 (3.2) | 10.9 (3.2) | 10.8 (3.0) | 10.4 (3.3) | .14 |  |
| **Latest pTau217 range**^§^ |  |  |  |  | .20 |  |
| *pTau217 Low* | 651 (60%) | 260 (59%) | 183 (60%) | 208 (62%) |  |  |
| *pTau217 Moderate* | 227 (21%) | 84 (19%) | 67 (22%) | 76 (22%) |  |  |
| *pTau217 High* | 209 (19%) | 98 (22%) | 57 (19%) | 54 (16%) |  |  |
| Values are Mean (SD) or No. (%), unless otherwise noted.  ^*^Representative of baseline LIBRA score (defined as first full LIBRA score). Low risk (-4.2 – 0.1), moderate risk (0.2 – 1.9), high risk (2.0 – 7.9).  ^†^Statistical tests: χ^2^ or Fisher's exact test for categorical variables, Wilcoxon rank sum test for continuous variables. *P* value for difference between baseline LIBRA tertiles; pairwise post hoc differences are reported for groups with *P* <.05.  **^‡^**Participants can report multiple races. Representative of reporting instances.  ^§^Plasma pTau217-ALZpath cut-offs at 0.40 pg/mL and 0.63 pg/mL.  Abbreviations: LIBRA, Lifestyle for BRAin health index; APOE, apolipoprotein E; CU, cognitively unimpaired; MCI, mild cognitive impairment; WRAT-3, Wide Range Achievement Test (3rd edition); PACC-3, Preclinical Alzheimer's Cognitive Composite (three-test version with Digit Symbol). | | | | | | |

**Supplementary Table 3. Risk of incident cognitive impairment according to baseline LIBRA tertiles, *APOE*𝜀4 carriage, and the interaction of the two (across all definitions of incident cognitive impairment)**

|  | | **Incident cognitive impairment^*^** | | | | | | | |
| --- | --- | --- | --- | --- | --- | --- | --- | --- | --- |
|  |  | Defined as PACC-3 performance below 7^th^ percentile on cross-sectional internal PACC-3 norms (adjusted for age, sex, education, and WRAT-3) | | Defined as two consecutive PACC-3 performances below 7^th^ percentile on cross-sectional internal PACC-3 norms (adjusted for age, sex, education, and WRAT-3) | | Defined as PACC-3 performance below 7^th^ percentile longitudinal internal PACC-3 norms (adjusted for age, sex, education, WRAT-3, practice, and baseline PACC-3 performance) | | Defined as two consecutive PACC-3 performances below 7^th^ percentile longitudinal internal PACC-3 norms (adjusted for age, sex, education, WRAT-3, practice, and baseline PACC-3 performance) | |
|  |  | N, impaired = 278 | | N, impaired = 105 | | N, impaired = 304 | | N, impaired = 76 | |
| **Model^†^** | | **HR (95% CI)** | ***P*** | **HR (95% CI)** | ***P*** | **HR (95% CI)** | ***P*** | **HR (95% CI)** | ***P*** |
| 1 | **Baseline LIBRA** |  |  |  |  |  |  |  |  |
|  | *Low Risk* | 1 [Reference] |  | 1 [Reference] |  | 1 [Reference] |  | 1 [Reference] |  |
|  | *Moderate Risk* | 1.57 (1.16-2.12) | .004 | 1.19 (0.71-2.00) | .52 | 1.13 (0.86-1.50) | .37 | 0.86 (0.47-1.56) | .62 |
|  | *High Risk* | 2.10 (1.58-2.80) | <.001 | 2.24 (1.43-3.51) | <.001 | 1.33 (1.02-1.74) | .04 | 1.66 (0.99-2.78) | .05 |
| **Harrell C** | | *0.58* | | *0.61* | | *0.54* | | *0.56* | |
| 2 | **Baseline LIBRA** |  |  |  |  |  |  |  |  |
|  | *Low Risk* | 1 [Reference] |  | 1 [Reference] |  | 1 [Reference] |  | 1 [Reference] |  |
|  | *Moderate Risk* | 1.58 (1.17-2.15) | .003 | 1.22 (0.73-2.04) | .45 | 1.15 (0.87-1.51) | .34 | 0.90 (1.50-1.64) | .74 |
|  | *High Risk* | 2.08 (1.56-2.76) | <.001 | 2.22 (1.42-3.49) | <.001 | 1.32 (1.01-1.73) | .04 | 1.56 (0.99-2.77) | 0.56 |
|  | ***APOE*ε4** |  |  |  |  |  |  |  |  |
|  | *ε4 Non-carrier* | 1 [Reference] |  | 1 [Reference] |  | 1 [Reference] |  | 1 [Reference] |  |
|  | *ε4 Carrier* | 1.89 (1.49-2.39) | <.001 | 2.51 (1.71-3.71) | <.001 | 1.82 (1.45-2.29) | <.001 | 2.80 (1.77-4.42) | <.001 |
| **Harrell C** | | *0.61* | | *0.67* | | *0.59* | | *0.65* | |
| 3 | **Baseline LIBRA tertiles × *APOE*ε4** |  |  |  |  |  |  |  |  |
|  | *Low Risk,*  *ε4 Non-carrier* | 1 [Reference] |  | 1 [Reference] |  | 1 [Reference] |  | 1 [Reference] |  |
|  | *Moderate Risk,*  *ε4 Carrier* | 0.67 (0.36-1.21) | .18 | 1.38 (0.47-4.01) | .56 | 0.86 (0.49-1.49) | .58 | 0.86 (0.25-2.93) | .81 |
|  | *High Risk,*  *ε4 Carrier* | 0.70 (0.34-1.06) | .08 | 0.50 (0.20-1.24) | .14 | 0.75 (0.44-1.28) | .28 | 0.37 (0.13-1.07) | .07 |
| **Harrell C** | | *0.61* | | *0.68* | | *0.59* | | *0.65* | |
| **LRT of Models 2 & 3** | | 𝜒^2^(2)=3.38, p=.18 | | 𝜒^2^(2)=4.71, p=.09 | | 𝜒^2^(2)=1.16, p=.56 | | 𝜒^2^(2)=3.83, p=.15 | |
| ^*^The PACC-3 cross-sectional and longitudinal norms were determined using quantile regression, extending the methods described by Jonaitis et al. [37, 41]. Individual percentile ranks were estimated from each model, and incident cognitive impairment was classified as the first visit where PACC-3 performance was below the 7th percentile.  ^†^Model 1: Cox regression model of incident cognitive impairment according to baseline LIBRA tertile with age as the time scale. Model 2: Cox regression model of incident cognitive impairment according to baseline LIBRA tertile and *APOE*ε4 carriage with age as the time scale. Model 3: Cox regression model of incident cognitive impairment according to the interaction of baseline LIBRA tertile and *APOE*ε4 carriage with age as the time scale.  Abbreviations: LIBRA, Lifestyle for Brain Health Index; APOE, apolipoprotein E; PACC-3, Three-test Preclinical Alzheimer Cognitive Composite; WRAT-3, Wide Range Achievement Test (3rd edition); HR, hazard ratio; CI, confidence interval. | | | | | | | | | |

**Supplementary Table 4. Risk of incident cognitive impairment according to baseline continuous LIBRA, *APOE*𝜀4 carriage, and the interaction of the two (across all definitions of incident cognitive impairment)**

|  | | **Incident cognitive impairment^*^** | | | | | | | |  |
| --- | --- | --- | --- | --- | --- | --- | --- | --- | --- | --- |
|  |  | Defined as PACC-3 performance below 7^th^ percentile on cross-sectional internal PACC-3 norms (adjusted for age, sex, education, and WRAT-3) | | Defined as two consecutive PACC-3 performances below 7^th^ percentile on cross-sectional internal PACC-3 norms (adjusted for age, sex, education, and WRAT-3) | | Defined as PACC-3 performance below 7^th^ percentile longitudinal internal PACC-3 norms (adjusted for age, sex, education, WRAT-3, practice, and baseline PACC-3 performance) | | Defined as two consecutive PACC-3 performances below 7^th^ percentile longitudinal internal PACC-3 norms (adjusted for age, sex, education, WRAT-3, practice, and baseline PACC-3 performance) | |  |
|  |  | N, impaired = 278 | | N, impaired = 105 | | N, impaired = 304 | | N, impaired = 76 | |  |
| **Model^†^** | | **HR (95% CI)** | ***P*** | **HR (95% CI)** | ***P*** | **HR (95% CI)** | ***P*** | **HR (95% CI)** | ***P*** |  |
| 1 | **Baseline LIBRA** | 1.18 (1.12-1.24) | <.001 | 1.20 (1.10-1.30) | <.001 | 1.08 (1.03-1.13) | .003 | 1.10 (0.99-1.21) | .08 |  |
| **Harrell C** | | *0.58* | | *0.61* | | *0.54* | | *0.56* | |  |
| 2 | **Baseline LIBRA** | 1.18 (1.12-1.24) | <.001 | 1.19 (1.09-1.30) | <.001 | 1.07 (1.02-1.13) | .006 | 1.09 (0.98-1.21) | .10 |  |
|  | ***APOE*ε4** |  |  |  |  |  |  |  |  |  |
|  | *ε4 Non-carrier* | 1 [Reference] |  | 1 [Reference] |  | 1 [Reference] |  | 1 [Reference] |  |  |
|  | *ε4 Carrier* | 1.85 (1.47-2.35) | <.001 | 2.49 (1.69-3.66) | <.001 | 1.80 (1.44-2.27) | <.001 | 2.81 (1.78-4.44) | <.001 |  |
| **Harrell C** | | *0.63* | | *0.68* | | *0.59* | | *0.65* | |  |
| 3 | **Baseline LIBRA × *APOE*ε4** |  |  |  |  |  |  |  |  |  |
|  | *Baseline LIBRA, ε4 Non-carrier* | 1 [Reference] |  | 1 [Reference] |  | 1 [Reference] |  | 1 [Reference] |  |  |
|  | *Baseline LIBRA, ε4 Carrier* | 0.95 (0.85-1.06) | .34 | 0.87 (0.73-1.03) | .11 | 0.98 (0.89-1.09) | .74 | 0.87 (0.71-1.07) | .20 |  |
| **Harrell C** | | *0.63* | | *0.69* | | *0.59* | | *0.65* | |  |
| **LRT of Models 2 & 3** | | 𝜒^2^(1)=0.92, p=.34 | | 𝜒^2^(1)=2.50, p=.11 | | 𝜒^2^(1)=0.11, p=.74 | | 𝜒^2^(1)=1.67, p=.20 | |  |
| ^*^The PACC-3 cross-sectional and longitudinal norms were determined using quantile regression, using the methods described by Jonaitis et al. [37, 41]. Individual percentile ranks were estimated from each model, and incident cognitive impairment was classified as the first visit where PACC-3 performance was below the 7th percentile.  ^†^Model 1: Cox regression model of incident cognitive impairment according to continuous baseline LIBRA with age as the time scale. Model 2: Cox regression model of incident cognitive impairment according to continuous baseline LIBRA and *APOE*ε4 carriage with age as the time scale. Model 3: Cox regression model of incident cognitive impairment according to the interaction of continuous baseline LIBRA and *APOE*ε4 carriage with age as the time scale.  Abbreviations: LIBRA, Lifestyle for Brain Health Index; APOE, apolipoprotein E; PACC-3, Three-test Preclinical Alzheimer Cognitive Composite; WRAT-3, Wide Range Achievement Test (3rd edition); HR, hazard ratio; CI, confidence interval. | | | | | | | | | | |

**Supplementary Table 5. Risk of incident cognitive impairment according to baseline LIBRA tertile and *APOE*ε4 carriage combined six-level factor (across all definitions of incident cognitive impairment)**

|  | **Incident cognitive impairment^*^** | | | | | | | |
| --- | --- | --- | --- | --- | --- | --- | --- | --- |
|  | Defined as PACC-3 performance below 7^th^ percentile on cross-sectional internal PACC-3 norms (adjusted for age, sex, education, and WRAT-3) | | Defined as two consecutive PACC-3 performances below 7^th^ percentile on cross-sectional internal PACC-3 norms (adjusted for age, sex, education, and WRAT-3) | | Defined as PACC-3 performance below 7^th^ percentile longitudinal internal PACC-3 norms (adjusted for age, sex, education, WRAT-3, practice, and baseline PACC-3 performance) | | Defined as two consecutive PACC-3 performances below 7^th^ percentile longitudinal internal PACC-3 norms (adjusted for age, sex, education, WRAT-3, practice, and baseline PACC-3 performance) | |
|  | N, impaired = 278 | | N, impaired = 105 | | N, impaired = 304 | | N, impaired = 76 | |
| **Model terms^†^** | **HR (95% CI)** | ***P*** | **HR (95% CI)** | ***P*** | **HR (95% CI)** | ***P*** | **HR (95% CI)** | ***P*** |
| ***APOE*𝜀4 non-carrier** |  |  |  |  |  |  |  |  |
| *Low Risk* | 1 [Reference] |  | 1 [Reference] |  | 1 [Reference] |  | 1 [Reference] |  |
| *Moderate Risk* | 1.97 (1.27 – 3.03) | .002 | 1.01 (0.43-2.36) | .98 | 1.24 (0.84-1.81) | .28 | 1.01 (0.38-2.65) | .98 |
| *High Risk* | 2.71 (1.79 – 4.10) | <.001 | 3.22 (1.65-6.32) | <.001 | 1.52 (1.05-2.20) | .03 | 2.88 (1.30-6.36) | .01 |
| ***APOE*ε4 carrier** |  |  |  |  |  |  |  |  |
| *Low Risk* | 2.63 (1.70-4.07) | <.001 | 3.17 (1.57-6.38) | .001 | 2.10 (1.46-3.02) | <.001 | 4.26 (1.99-9.11) | <.001 |
| *Moderate Risk* | 3.40 (2.15-5.37) | <.001 | 4.40 (2.12-9.10) | <.001 | 2.22 (1.47-3.33) | .001 | 3.96 (1.53-8.91) | .004 |
| *High Risk* | 4.25 (2.77-6.52) | <.001 | 5.12 (2.55-10.28) | <.001 | 2.38 (1.61-3.53) | <.001 | 4.57 (1.99-10.50) | <.001 |
| **Harrell C** | *0.61* | | *0.68* | | *0.59* | | *0.65* | |
| ^*^The PACC-3 cross-sectional and longitudinal norms were determined using quantile regression, using the methods described by Jonaitis et al. [37, 41]. Individual percentile ranks were estimated from each model, and incident cognitive impairment was classified as the first visit where PACC-3 performance was below the 7th percentile.  ^†^Cox regression model of incident cognitive impairment according to the combined six-level factor of baseline LIBRA tertiles and *APOE*ε4 carriage with age as the time scale*.*  Abbreviations: LIBRA, Lifestyle for Brain Health Index; APOE, apolipoprotein E; PACC-3, Three-test Preclinical Alzheimer Cognitive Composite; WRAT-3, Wide Range Achievement Test (3rd edition); HR, hazard ratio; CI, confidence interval. | | | | | | | | |

**Supplementary Table 6. Risk of incident cognitive impairment according to baseline LIBRA tertiles, plasma pTau217-ALZpath range, and the interaction of the two (across all definitions of incident cognitive impairment)**

|  | | **Incident cognitive impairment^*^** | | | | | | | |  |
| --- | --- | --- | --- | --- | --- | --- | --- | --- | --- | --- |
|  |  | Defined as PACC-3 performance below 7^th^ percentile on cross-sectional internal PACC-3 norms (adjusted for age, sex, education, and WRAT-3) | | Defined as two consecutive PACC-3 performances below 7^th^ percentile on cross-sectional internal PACC-3 norms (adjusted for age, sex, education, and WRAT-3) | | Defined as PACC-3 performance below 7^th^ percentile longitudinal internal PACC-3 norms (adjusted for age, sex, education, WRAT-3, practice, and baseline PACC-3 performance) | | Defined as two consecutive PACC-3 performances below 7^th^ percentile longitudinal internal PACC-3 norms (adjusted for age, sex, education, WRAT-3, practice, and baseline PACC-3 performance) | |  |
|  |  | N, impaired = 278 | | N, impaired = 105 | | N, impaired = 304 | | N, impaired = 76 | |  |
| **Model^†^** | | **HR (95% CI)** | ***P*** | **HR (95% CI)** | ***P*** | **HR (95% CI)** | ***P*** | **HR (95% CI)** | ***P*** |  |
| 1 | **Baseline LIBRA** |  |  |  |  |  |  |  |  |  |
|  | *Low Risk* | 1 [Reference] |  | 1 [Reference] |  | 1 [Reference] |  | 1 [Reference] |  |  |
|  | *Moderate Risk* | 1.57 (1.16-2.12) | .004 | 1.19 (0.71-2.00) | .52 | 1.14 (0.86-1.50) | .37 | 0.86 (0.47-1.56) | .62 |  |
|  | *High Risk* | 2.10 (1.58-2.80) | <.001 | 2.24 (1.43-3.51) | <.001 | 1.34 (1.02-1.75) | .03 | 1.66 (0.99-2.78) | .05 |  |
| **Harrell C** | | *0.58* | | *0.61* | | *0.54* | | *0.56* | |  |
| 2 | **Baseline LIBRA** |  |  |  |  |  |  |  |  |  |
|  | *Low Risk* | 1 [Reference] |  | 1 [Reference] |  | 1 [Reference] |  | 1 [Reference] |  |  |
|  | *Moderate Risk* | 1.63 (1.20-2.21) | .002 | 1.26 (0.75-2.12) | .37 | 1.18 (0.89-1.56) | .25 | 0.97 (0.53-1.77) | .93 |  |
|  | *High Risk* | 2.19 (1.64-2.91) | <.001 | 2.40 (1.53-3.78) | .001 | 1.39 (1.06-1.81) | .02 | 1.85 (1.10-3.10) | .02 |  |
|  | **pTau217** |  |  |  |  |  |  |  |  |  |
|  | *pTau217 Low* | 1 [Reference] |  | 1 [Reference] |  | 1 [Reference] |  | 1 [Reference] |  |  |
|  | *pTau217 Moderate* | 0.86 (0.63-1.19) | .37 | 1.03 (0.60-1.76) | .92 | 0.85 (0.63-1.16) | .31 | 0.45 (0.19-1.09) | .08 |  |
|  | *pTau217 High* | 1.56 (1.19-2.04) | .001 | 2.35 (1.53-3.59) | <.001 | 1.53 (1.18-1.98) | .001 | 2.78 (1.73-4.48) | <.001 |  |
| **Harrell C** | | *0.59* | | *0.64* | | *0.56* | | *0.67* | |  |
| 3**^‡^** | **Baseline LIBRA tertiles × pTau217** | 0.92 (0.78-1.08) | .29 | 0.83 (0.64-1.07) | .15 | 0.91 (0.78-1.06) | .22 | 0.82 (0.62-1.08) | .16 |  |
| **Harrell C** | | *0.59* | | *0.64* | | *0.57* | | *0.65* | |  |
| **LRT of Models 2 & 3** | | 𝜒^2^(4)=1.79, p=.77 | | 𝜒^2^(4)=7.90, p=.10 | | 𝜒^2^(4)=3.53, p=.47 | | 𝜒^2^(4)=5.31, p=.26 | |  |
| ^*^The PACC-3 cross-sectional and longitudinal norms were determined using quantile regression, extending the methods described by Jonaitis et al. [37, 41]. Individual percentile ranks were estimated from each model, and incident cognitive impairment was classified as the first visit where PACC-3 performance was below the 7th percentile.  ^†^Model 1: Cox regression model of incident cognitive impairment according to baseline LIBRA tertile with age as the time scale. Model 2: Cox regression model of incident cognitive impairment according to baseline LIBRA tertile and plasma pTau217-ALZpath range with age as the time scale. Model 3: Cox regression model of incident cognitive impairment according to the interaction of baseline LIBRA tertile and plasma pTau217-ALZpath range with age as the time scale.  **^‡^**Only the combined interaction term is reported here for ease of interpretability of the 3x3 interaction.  Abbreviations: LIBRA, Lifestyle for Brain Health Index; APOE, apolipoprotein E; PACC-3, Three-test Preclinical Alzheimer Cognitive Composite; WRAT-3, Wide Range Achievement Test (3rd edition); HR, hazard ratio; CI, confidence interval. | | | | | | | | | | |

**Supplementary Table 7. Risk of incident cognitive impairment according to baseline continuous LIBRA, plasma pTau217-ALZpath range, and the interaction of the two (across all definitions of incident cognitive impairment)**

|  | | **Incident cognitive impairment^*^** | | | | | | | |  |
| --- | --- | --- | --- | --- | --- | --- | --- | --- | --- | --- |
|  |  | Defined as PACC-3 performance below 7^th^ percentile on cross-sectional internal PACC-3 norms (adjusted for age, sex, education, and WRAT-3) | | Defined as two consecutive PACC-3 performances below 7^th^ percentile on cross-sectional internal PACC-3 norms (adjusted for age, sex, education, and WRAT-3) | | Defined as PACC-3 performance below 7^th^ percentile longitudinal internal PACC-3 norms (adjusted for age, sex, education, WRAT-3, practice, and baseline PACC-3 performance) | | Defined as two consecutive PACC-3 performances below 7^th^ percentile longitudinal internal PACC-3 norms (adjusted for age, sex, education, WRAT-3, practice, and baseline PACC-3 performance) | |  |
|  |  | N, impaired = 278 | | N, impaired = 105 | | N, impaired = 304 | | N, impaired = 76 | |  |
| **Model^†^** | | **HR (95% CI)** | ***P*** | **HR (95% CI)** | ***P*** | **HR (95% CI)** | ***P*** | **HR (95% CI)** | ***P*** |  |
| 1 | **Baseline LIBRA** | 1.18 (1.12-1.25) | <.001 | 1.20 (1.10-1.31) | <.001 | 1.08 (1.03-1.13) | .003 | 1.10 (0.99-1.21) | .08 |  |
| **Harrell C** | | *0.60* | | *0.63* | | *0.56* | | *0.56* | |  |
| 2 | **Baseline LIBRA** | 1.19 (1.13-1.25) | <.001 | 1.21 (1.11-1.31) | <.001 | 1.08 (1.03-1.14) | .002 | 1.11 (1.01-1.23) | .04 |  |
|  | **pTau217** |  |  |  |  |  |  |  |  |  |
|  | *pTau217 Low* | 1 [Reference] |  | 1 [Reference] |  | 1 [Reference] |  | 1 [Reference] |  |  |
|  | *pTau217 Moderate* | 0.86 (0.63-1.19) | .37 | 1.02 (0.59-1.75) | .95 | 0.85 (0.63-1.16) | .30 | 0.45 (0.19-1.07) | .07 |  |
|  | *pTau217 High* | 1.54 (1.18-2.02) | .002 | 2.32 (1.52-3.55) | <.001 | 1.52 (1.18-1.97) | .001 | 2.76 (1.72-4.44) | <.001 |  |
| **Harrell C** | | *0.62* | | *0.65* | | *0.58* | | *0.66* | |  |
| 3 | **Baseline LIBRA × pTau217** |  |  |  |  |  |  |  |  |  |
|  | *Baseline LIBRA, pTau217 Low* | 1 [Reference] |  | 1 [Reference] |  | 1 [Reference] |  | 1 [Reference] |  |  |
|  | *Baseline LIBRA, pTau217 Moderate* | 0.94 (0.81-1.09) | 0.41 | 0.81 (0.63-1.03) | .08 | 0.97 (0.85-1.12) | .71 | 0.82 (0.55-1.21) | .31 |  |
|  | *Baseline LIBRA, pTau217 High* | 0.96 (0.85-1.08) | .46 | 0.81 (0.68-0.99) | .03 | 0.92 (0.83-1.03) | .16 | 0.81 (0.66-1.00) | .05 |  |
| **Harrell C** | | *0.62* | | *0.67* | | *0.58* | | *0.66* | |  |
| **LRT of Model 2 & 3** | | 𝜒^2^(2)=0.91, p=.64 | | 𝜒^2^(2)=5.38, p=.07 | | 𝜒^2^(2)=2.00, p=.37 | | 𝜒^2^(2)=4.00, p=.14 | |  |
| ^*^The PACC-3 cross-sectional and longitudinal norms were determined using quantile regression, using the methods described by Jonaitis et al. [37, 41]. Individual percentile ranks were estimated from each model, and incident cognitive impairment was classified as the first visit where PACC-3 performance was below the 7th percentile.  ^†^Model 1: Cox regression model of incident cognitive impairment according to continuous baseline LIBRA with age as the time scale. Model 2: Cox regression model of incident cognitive impairment according to continuous baseline LIBRA and plasma pTau217-ALZpath range with age as the time scale. Model 3: Cox regression model of incident cognitive impairment according to the interaction of continuous baseline LIBRA and plasma pTau217-ALZpath range with age as the time scale.  Abbreviations: LIBRA, Lifestyle for Brain Health Index; APOE, apolipoprotein E; PACC-3, Three-test Preclinical Alzheimer Cognitive Composite; WRAT-3, Wide Range Achievement Test (3rd edition); HR, hazard ratio; CI, confidence interval. | | | | | | | | | | |

**Supplementary Table 8. Risk of incident cognitive impairment according to baseline LIBRA tertile and plasma pTau217-ALZpath ranges combined nine-level factor (across all definitions of incident cognitive impairment)**

|  | **Incident cognitive impairment^*^** | | | | | | | |
| --- | --- | --- | --- | --- | --- | --- | --- | --- |
|  | Defined as PACC-3 performance below 7^th^ percentile on cross-sectional internal PACC-3 norms (adjusted for age, sex, education, and WRAT-3) | | Defined as two consecutive PACC-3 performances below 7^th^ percentile on cross-sectional internal PACC-3 norms (adjusted for age, sex, education, and WRAT-3) | | Defined as PACC-3 performance below 7^th^ percentile longitudinal internal PACC-3 norms (adjusted for age, sex, education, WRAT-3, practice, and baseline PACC-3 performance) | | Defined as two consecutive PACC-3 performances below 7^th^ percentile longitudinal internal PACC-3 norms (adjusted for age, sex, education, WRAT-3, practice, and baseline PACC-3 performance) | |
|  | N, impaired = 278 | | N, impaired = 105 | | N, impaired = 304 | | N, impaired = 76 | |
| **Model terms^†^** | **HR (95% CI)** | ***P*** | **HR (95% CI)** | ***P*** | **HR (95% CI)** | ***P*** | **HR (95% CI)** | ***P*** |
| **pTau217 Low** |  |  |  |  |  |  |  |  |
| *Low Risk* | 1 [Reference] |  | 1 [Reference] |  | 1 [Reference] |  | 1 [Reference] |  |
| *Moderate Risk* | 1.90 (1.22-2.96) | .005 | 0.81 (0.30-2.18) | .67 | 1.23 (0.82-1.85) | .32 | 0.84 (0.28-2.50) | .75 |
| *High Risk* | 2.66 (1.75-4.03) | <.001 | 3.62 (1.78-7.34) | <.001 | 1.69 (1.15-2.47) | .007 | 3.06 (1.36-6.88) | .007 |
| **pTau217 Moderate** |  |  |  |  |  |  |  |  |
| *Low Risk* | 1.16 (0.63-2.13) | .63 | 1.45 (0.54-3.93) | .46 | 1.10 (0.67-1.82) | .70 | 0.88 (0.24-3.27) | .85 |
| *Moderate Risk* | 1.57 (0.88-2.81) | .13 | 1.98 (0.77-5.11) | .16 | 0.96 (0.55-1.69) | .89 | 0.67 (0.14-3.10) | .61 |
| *High Risk* | 1.96 (1.13-3.39) | .02 | 1.89 (0.70-5.14) | .21 | 1.20 (0.71-2.03) | .50 | 0.42 (0.05-3.31) | .41 |
| **pTau217 High** |  |  |  |  |  |  |  |  |
| *Low Risk* | 1.92 (1.19-3.09) | .007 | 2.75 (1.27-5.92) | .01 | 1.70 (1.13-2.55) | .01 | 3.65 (1.64-8.14) | .002 |
| *Moderate Risk* | 2.81 (1.67-4.73) | <.001 | 4.39 (1.96-9.84) | <.001 | 2.26 (1.43-3.57) | <.001 | 4.10 (1.56-10.10) | .002 |
| *High Risk* | 3.66 (2.24-5.96) | <.001 | 5.19 (2.35-11.45) | <.001 | 1.93 (1.20-3.12) | .007 | 5.06 (2.09-12.24) | <.001 |
| **Harrell C** | *0.59* | | *0.66* | | *0.60* | | *0.68* | |
| ^*^The PACC-3 cross-sectional and longitudinal norms were determined using quantile regression, using the methods described by Jonaitis et al. [37, 41]. Individual percentile ranks were estimated from each model, and incident cognitive impairment was classified as the first visit where PACC-3 performance was below the 7th percentile.  ^†^Cox regression model of incident cognitive impairment according to the combined nine-level factor of LIBRA tertiles and plasma pTau217-ALZpath ranges with age as the time scale*.*  Abbreviations: LIBRA, Lifestyle for Brain Health Index; APOE, apolipoprotein E; PACC-3, Three-test Preclinical Alzheimer Cognitive Composite; WRAT-3, Wide Range Achievement Test (3rd edition); HR, hazard ratio; CI, confidence interval. | | | | | | | | |

**Supplementary Figure 1: Participant inclusion/exclusion flow diagram**

**Supplementary Figure 2.** Cox proportional hazard model output (panel A) and survival curves (panel B) based on ICI as defined by PACC-3 performance below 7th percentile on longitudinal internal PACC-3 norms (adjusted for age, sex, education, WRAT-3, practice, and baseline PACC-3 performance). Model 1: baseline LIBRA risk tertile; Model 2: baseline LIBRA risk tertile and *APOE*ε4 carriage.

**Supplementary Figure 3.** Cox proportional hazard model output (panel A) and survival curves (panel B) based on ICI as defined by PACC-3 performance below 7th percentile on two consecutive cross-sectional internal PACC-3 norms (adjusted for age, sex, education, WRAT-3, practice, and baseline PACC-3 performance). Model 1: baseline LIBRA risk tertile; Model 2: baseline LIBRA risk tertile and *APOE*ε4 carriage.

**Supplementary Figure 4.** Cox proportional hazard model output (panel A) and survival curves (panel B) based on ICI as defined by PACC-3 performance below 7th percentile on two consecutive longitudinal internal PACC-3 norms (adjusted for age, sex, education, WRAT-3, practice, and baseline PACC-3 performance). Model 1: baseline LIBRA risk tertile; Model 2: baseline LIBRA risk tertile and *APOE*ε4 carriage.

**Supplementary Figure 5.** Cox proportional hazard model output (panel A) and survival curves (panel B), based on ICI as defined by PACC-3 performance below 7th percentile on longitudinal internal PACC-3 norms (adjusted for age, sex, education, and WRAT-3), for a six-level combination of baseline LIBRA tertile and *APOE*ε4 carriage (6 categories with low lifestyle risk, *APOE*ε4 non-carriers as the reference).

**Supplementary Figure 6.** Cox proportional hazard model output (panel A) and survival curves (panel B), based on ICI as defined by PACC-3 performance below 7th percentile on two consecutive cross-sectional internal PACC-3 norms (adjusted for age, sex, education, and WRAT-3), for a six-level combination of baseline LIBRA tertile and *APOE*ε4 carriage (6 categories with low lifestyle risk, *APOE*ε4 non-carriers as the reference).

**Supplementary Figure 7.** Cox proportional hazard model output (panel A) and survival curves (panel B), based on ICI as defined by PACC-3 performance below 7th percentile on two consecutive longitudinal internal PACC-3 norms (adjusted for age, sex, education, and WRAT-3), for a six-level combination of baseline LIBRA tertile and *APOE*ε4 carriage (6 categories with low lifestyle risk, *APOE*ε4 non-carriers as the reference).

**Supplementary Figure 8.** Cox proportional hazard model output (panel A) and survival curves (panel B) based on ICI as defined by PACC-3 performance below 7th percentile on longitudinal internal PACC-3 norms (adjusted for age, sex, education, WRAT-3, practice, and baseline PACC-3 performance). Model 1: baseline LIBRA risk tertile; Model 2: baseline LIBRA risk tertile and plasma pTau217-ALZpath range.

**Supplementary Figure 9.** Cox proportional hazard model output (panel A) and survival curves (panel B) based on ICI as defined by PACC-3 performance below 7th percentile on two consecutive cross-sectional internal PACC-3 norms (adjusted for age, sex, education, WRAT-3, practice, and baseline PACC-3 performance). Model 1: baseline LIBRA risk tertile; Model 2: baseline LIBRA risk tertile and plasma pTau217-ALZpath range.

**Supplementary Figure 10.** Cox proportional hazard model output (panel A) and survival curves (panel B) based on ICI as defined by PACC-3 performance below 7th percentile on two consecutive longitudinal internal PACC-3 norms (adjusted for age, sex, education, WRAT-3, practice, and baseline PACC-3 performance). Model 1: baseline LIBRA risk tertile; Model 2: baseline LIBRA risk tertile and plasma pTau217-ALZpath range.

**Supplementary Figure 11.** Cox proportional hazard model output (panel A) and survival curves (panels B-D), based on ICI as defined by PACC-3 performance below 7th percentile on longitudinal internal PACC-3 norms (adjusted for age, sex, education, and WRAT-3), for a nine-level combination of baseline LIBRA tertile and plasma pTau217-ALZpath range (9 categories with low lifestyle risk, pTau217 low as the reference).

**Supplementary Figure 12.** Cox proportional hazard model output (panel A) and survival curves (panels B-D), based on ICI as defined by PACC-3 performance below 7th percentile on two consecutive cross-sectional internal PACC-3 norms (adjusted for age, sex, education, and WRAT-3), for a nine-level combination of baseline LIBRA tertile and plasma pTau217-ALZpath range (9 categories with low lifestyle risk, pTau217 low as the reference).

**Supplementary Figure 13.** Cox proportional hazard model output (panel A) and survival curves (panels B-D), based on ICI as defined by PACC-3 performance below 7th percentile on two consecutive longitudinal internal PACC-3 norms (adjusted for age, sex, education, and WRAT-3), for a nine-level combination of baseline LIBRA tertile and plasma pTau217-ALZpath range (9 categories with low lifestyle risk, pTau217 low as the reference).
